# Die Chefin des Nobelpreisträgers: Elsbet Forßmann (1906–1993), geb. Engel

**DOI:** 10.1007/s00120-026-02908-2

**Published:** 2026-09-30

**Authors:** Julia Nebe, Friedrich Moll, Matthis Krischel

**Affiliations:** 1 Institut für Geschichte, Theorie und Ethik der Medizin Heinrich-Heine-Universität Düsseldorf, Moorenstr. 5, 40225 Düsseldorf, Deutschland; 2 Universitätsklinikum Düsseldorf. Anstalt öffentlichen Rechts, Düsseldorf, Deutschland

**Keywords:** Medizingeschichte, Geschichte der Urologie, Erinnerungskultur, Nationalsozialismus, Urologinnen, History of medicine, History of urology, Culture of remembrance, National Socialism, Women in urology

## Abstract

Lange Zeit haftete Elsbet Forßmann (1906–1993) das Narrativ der „tapferen und verständnisvollen Gattin“ des Nobelpreisträgers Werner Forßmann (1904–1979) an. Doch sie war weit mehr als das. Nach ihrer Ausbildung bei Karl Heusch (1894–1986) am Rudolf-Virchow-Krankenhaus in Berlin in den Jahren 1933 bis 1934 gehörte sie zu den beiden ersten Ärztinnen, die in der Nachkriegszeit den Facharzt für Urologie erwarben. Auf Grundlage einer breiten chirurgischen Ausbildung deckte sie in ihrer praktischen Tätigkeit die „ganze Breite der Urologie“ ab und blieb ihrer Spezialisierung ein Leben lang verbunden. Als Mutter von 6 Kindern sicherte sie nach 1945 mit ihrer Landarztpraxis in Bad Kreuznach zeitweise die wirtschaftliche Existenz ihrer Familie; auch ihr Ehemann Werner Forßmann arbeitete in dieser Praxis. Elsbet Forßmann zählt damit zweifelsohne zu den Pionierinnen der bundesrepublikanischen Urologiegeschichte. Als in der öffentlichen und fachkulturellen Wahrnehmung lange Zeit auf die Rolle der „Frau des Nobelpreisträgers“ reduzierte Persönlichkeit ist die Auseinandersetzung mit Forßmanns Lebensweg untrennbar mit Fragen nach geschlechtlicher Zuschreibung wissenschaftlicher Anerkennungskulturen und fachkultureller Erinnerung verbunden. Der Beitrag zeigt, wie selektiv fachkulturelles Gedächtnis funktioniert und welchen Einfluss soziale Herkunft, Geschlecht sowie strukturelle Bedingungen wissenschaftlicher Anerkennung auf Sichtbarkeit und Vergessen in der Medizingeschichte haben. Die Beschäftigung mit Elsbet Forßmanns Biografie versteht sich damit zugleich als Beitrag zu einer kritisch-reflexiven Erinnerungskultur innerhalb der Urologie.

## Abstract (en)

This article highlights the life and work of the urologist Elsbet Forßmann (1906–1993), who was remembered for a very long time as the “brave and understanding wife” of Nobel Prize laureate Werner Forßmann (1904–1979) and draws attention to the widespread invisibility of women in the culture of memory of the field. But she was far more than that. After completing her training under Karl Heusch (1894–1986) at the Rudolf Virchow Hospital in Berlin from 1933–1934, she was one of the first two female physicians to earn board certification in urology in the postwar period. Building on broad surgical training, her clinical practice covered the “entire spectrum of urology,” and she remained dedicated to her specialty throughout her life. As the mother of six children, she temporarily ensured her family’s financial security after 1945 with her rural medical practice in Bad Kreuznach; her husband, Werner Forßmann, also worked at this practice. Elsbet Forßmann is thus undoubtedly one of the pioneers in the history of urology in the Federal Republic of Germany. As a figure long reduced in public and professional discourse to the role of the “wife of the Nobel laureate,” an examination of Forßmann’s life is inextricably linked to questions of gendered attributions within cultures of scientific recognition and professional memory within the histories of scientific disciplines. This article demonstrates how selectively disciplinary memory functions and how social background, gender, and the structural conditions of scientific recognition influence visibility and cultures of recognition in academia. This examination of Elsbet Forßmann’s biography can thus also be seen as a contribution to a culture of critical and reflective remembrance within the field of urology.

## Herkunft, Schulbildung und Studium

**Abb. 1:**
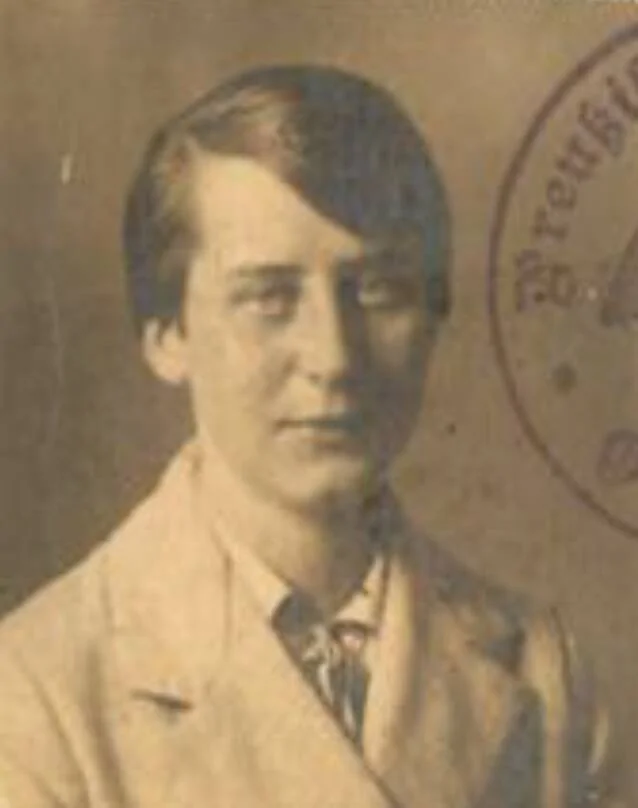
Elsbet Engel (WS1928/29)^a^. (Mit freundl. Genehmigung; ^a^UA Göttingen, Bestand: Abgangszeugnis 1930 Nr. 728)

Elisabeth Maria Margareta Klara Forßmann kurz Elsbet (geb. Engel) kam am 25. Februar 1906 als Tochter des protestantischen Theologen und Studienrates Heinrich Engel und seiner Ehefrau Berta (geb. Krieger) in Bingen am Rhein zur Welt. Nur 2 Jahre später folgt ihr ihr jüngerer Bruder Heinrich Karl Engel (26.04.1908; Abb. 1).^1^

Wie aus ihrem handschriftlichen Lebenslauf ihrer Personalakte des städtischen Krankenhauses in Mainz, dem heutigen Universitätsklinikum, hervorgeht, besuchte sie 10 Jahre lang die 1889 in Mainz neugegründete Höhere Mädchenschule, dem heutigen Frauenlob-Gymnasium, sowie die angegliederte Studienanstalt (für Lehrerinnen). Die Höhere Mädchenschule in Mainz war die erste staatliche höhere Schule für Mädchen, an der diese – je nach Altersstufe – gegen erhebliche Gebühren Unterricht erhalten konnten. Diese entsprachen teilweise einem ganzen Monatsgehalt eines durchschnittlichen Erwerbstätigen im Deutschen Reich zu dieser Zeit.^2^ Damit wird deutlich, dass höhere Bildung zu Beginn des 20. Jahrhunderts trotz staatlicher Bildungsangebote weiterhin sozial exklusiv war und insbesondere für Mädchen aus weniger privilegierten Familien nur eingeschränkt zugänglich blieb. [1, 2, 29, 30, 34, 36].

**Abb. 2:**
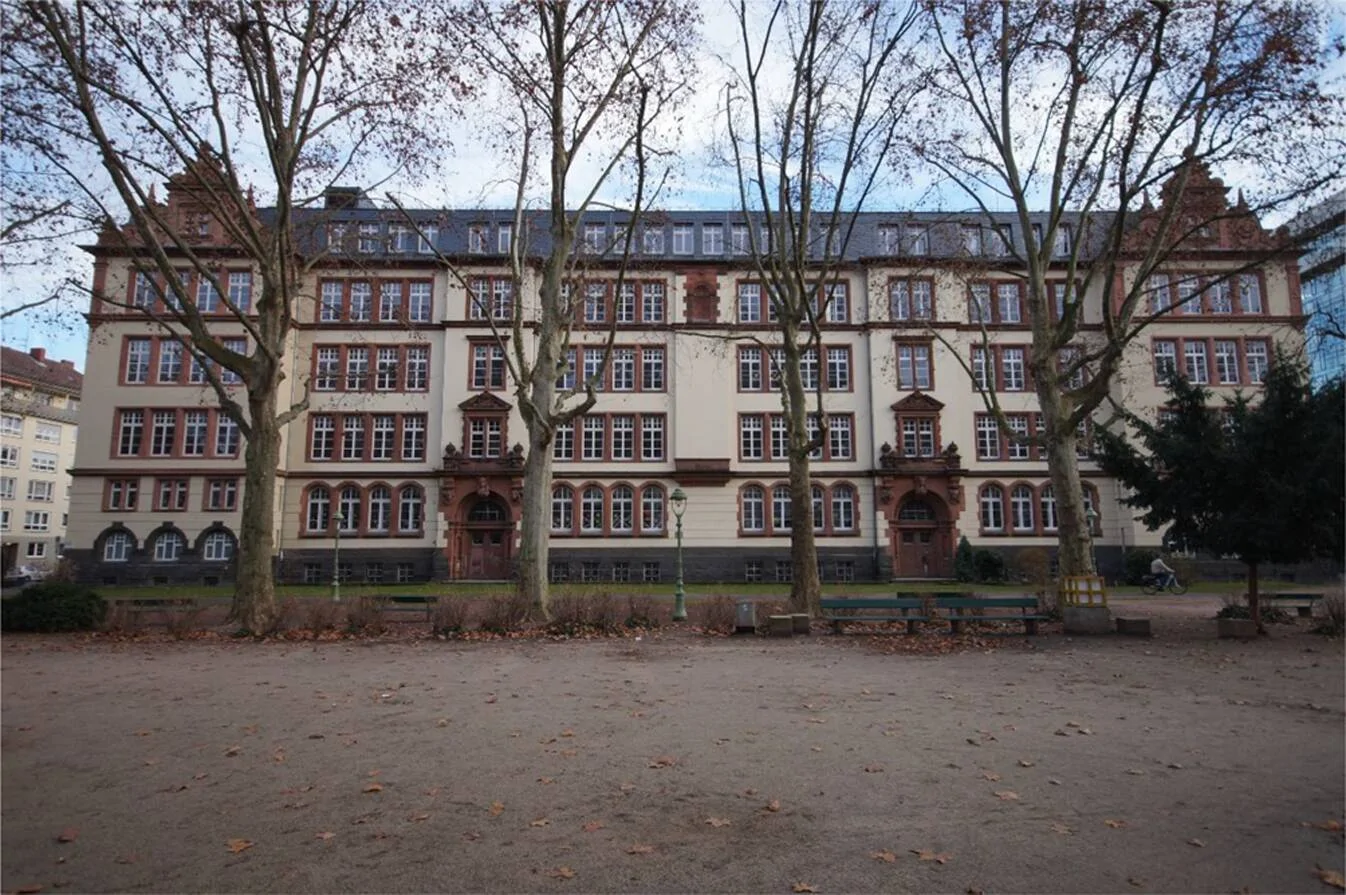
Die ehemalige Frauenarbeitsschule am Feldbergplatz 4, 2012^a^ (^a^Jivee Blau (2012) Mainz–Feldbergplatz–Feldbergschule 29.12.2012. JPG. Wikimedia Commons. Verfügbar unter: https://commons.wikimedia.org/wiki/File:Mainz-_Feldbergplatz-_Feldbergschule_29.12.2012.JPG. Zugegriffen: 20.07.2026.)

Der Besuch einer Höheren Mädchenschule war jedoch nicht mit dem Erwerb der Hochschulreife verbunden, wie dies an einem Mädchengymnasium möglich gewesen wäre. Stattdessen endete die Ausbildung mit der Reifeprüfung, die keinen Lateinunterricht einschloss [1, 2, 29, 30, 34, 36, 37, 41, 42]. Diese Reifeprüfung absolvierte Engel am 20. März 1925. Im Anschluss immatrikulierte sie sich am 29. April 1925 in Jena für das Studienfach der Medizin. Nach 4 präklinischen Semestern bestand sie dort im Herbst 1927 ihr ärztliches Vorexamen, nachdem sie zuvor die Ergänzungsprüfung in Latein an einem Realgymnasium ablegt hatte.^3^ Wahrscheinlich ist, dass sie zu diesem Zweck das heutige Gymnasium am Kurfürstlichen Schloss, welches 1831 als städtische Realschule eröffnet und ab 1884 zum Realgymnasium aufgewertet wurde, besuchte ([14]; Abb. 2).

**Abb. 3:**
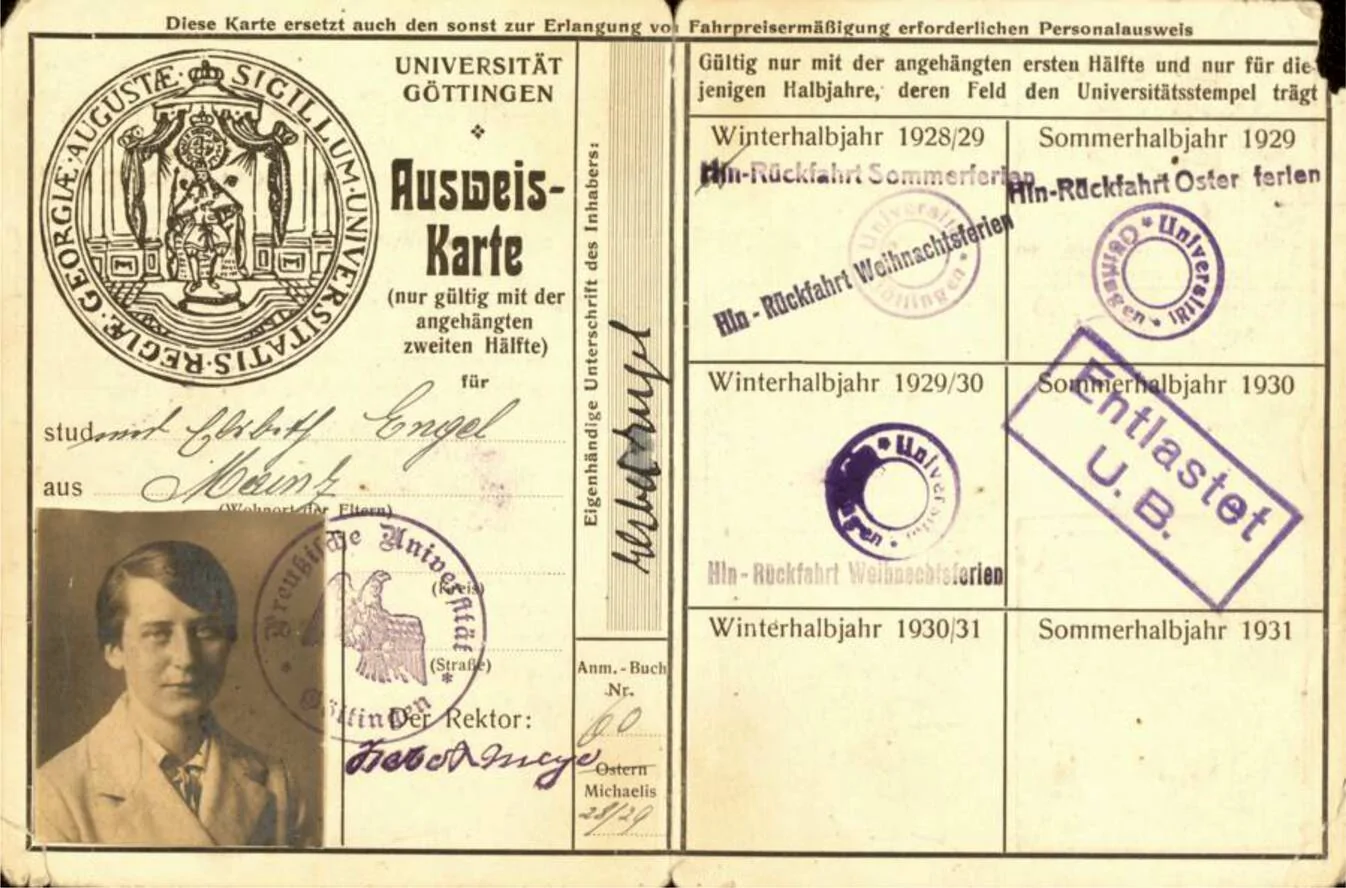
Elsbet Engel; Studierendenausweis Universität Göttingen (WS 1928/29 bis WS1929/30) ^a^. (Mit freundl. Genehmigung; ^a^UA Göttingen, Bestand: Abgangszeugnis 1930 Nr. 728.)

Nach dem Physikum setzte Engel ihr Medizinstudium – dem damals üblichen Wechsel zwischen verschiedenen Hochschulstandorten folgend – an mehreren Universitäten fort. Sie studierte zunächst im Wintersemester 1927/1928 in München, anschließend in Wien, wobei für diesen Studienabschnitt keine Studierendenakte überliefert ist, und wechselte schließlich an die Universität Göttingen. Dort war sie vom Wintersemester 1928/1929 bis zum Wintersemester 1929/1930 eingeschrieben und bestand am 30. Juli 1930 das medizinische Staatsexamen (Abb. 3). Der administrative Abschluss des Studiums zum Sommersemester 1930 wird durch den im Studierendenausweis überlieferten Vermerk „Entlastete U.B.“ [Universitätsbibliothek] bestätigt, der die vorschriftsmäßige Exmatrikulation am Tag des Examens besiegelt.^4^

**Abb. 4:**
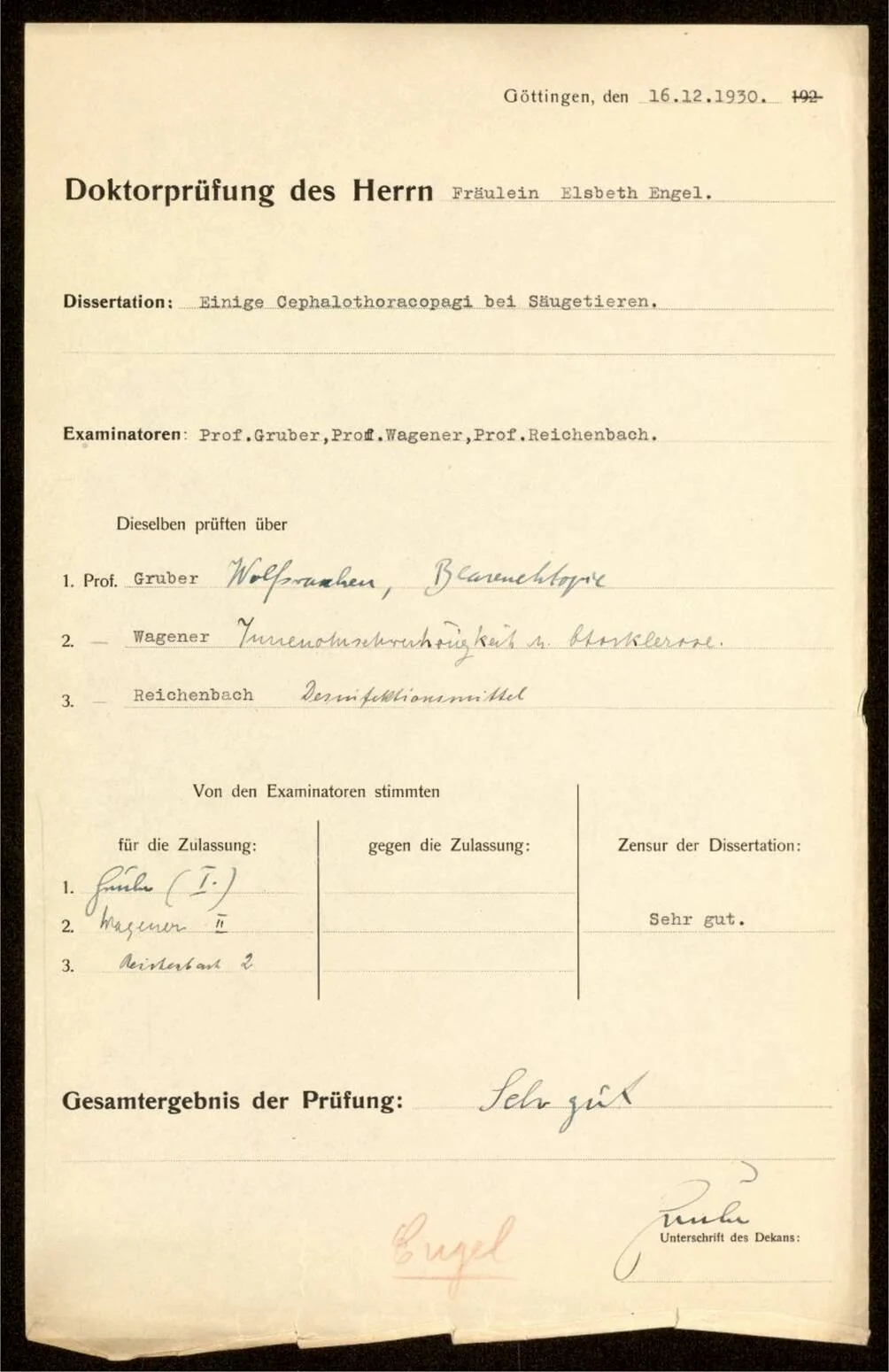
Zeugnis mündliches Doktorexamen, Göttingen 16.12.1930^a^. (Mit freundl. Genehmigung; ^a^UA Göttingen, Bestand: Abgangszeugnis 1930 Nr. 728.)

Ihre Promotion verfasste sie bei dem Pathologen Prof. Georg Benno Gruber (1884–1977) [23], der sein umfangreiches wissenschaftliches Werk u. a. auf dem Gebiet der Missbildungen hatte zum Thema „Einige Cephalothoracopagi bei Säugetieren“ [5, 6] am Pathologischen Institut in Göttingen. Ihr mündliches Doktorexamen bestand sie am 16.12.1930 mit der Note sehr gut; Prüfungsthemen waren u. a. Krankenhaushygiene, Fehlbildungen der Lippen und des Kiefers („Wolfsrachen“) sowie urogenitale Fehlbildungen, genauer Blasenekstrophie (Abb. 4).^5^

Elsbet Engel war damit eine von nur 3 Doktorandinnen, die in diesem Jahr, an der Medizinischen Fakultät der Universität Göttingen zum Dr. med. promoviert wurden [33]. Auffällig ist, dass auf dem Protokollbogen für den Prüfling die Anrede „Herr“ vorgedruckt ist – dies deutet auf die noch randständige Stellung von Doktorandinnen hin.^6^

## Internistische und pathologische Ausbildung

**Abb. 5:**
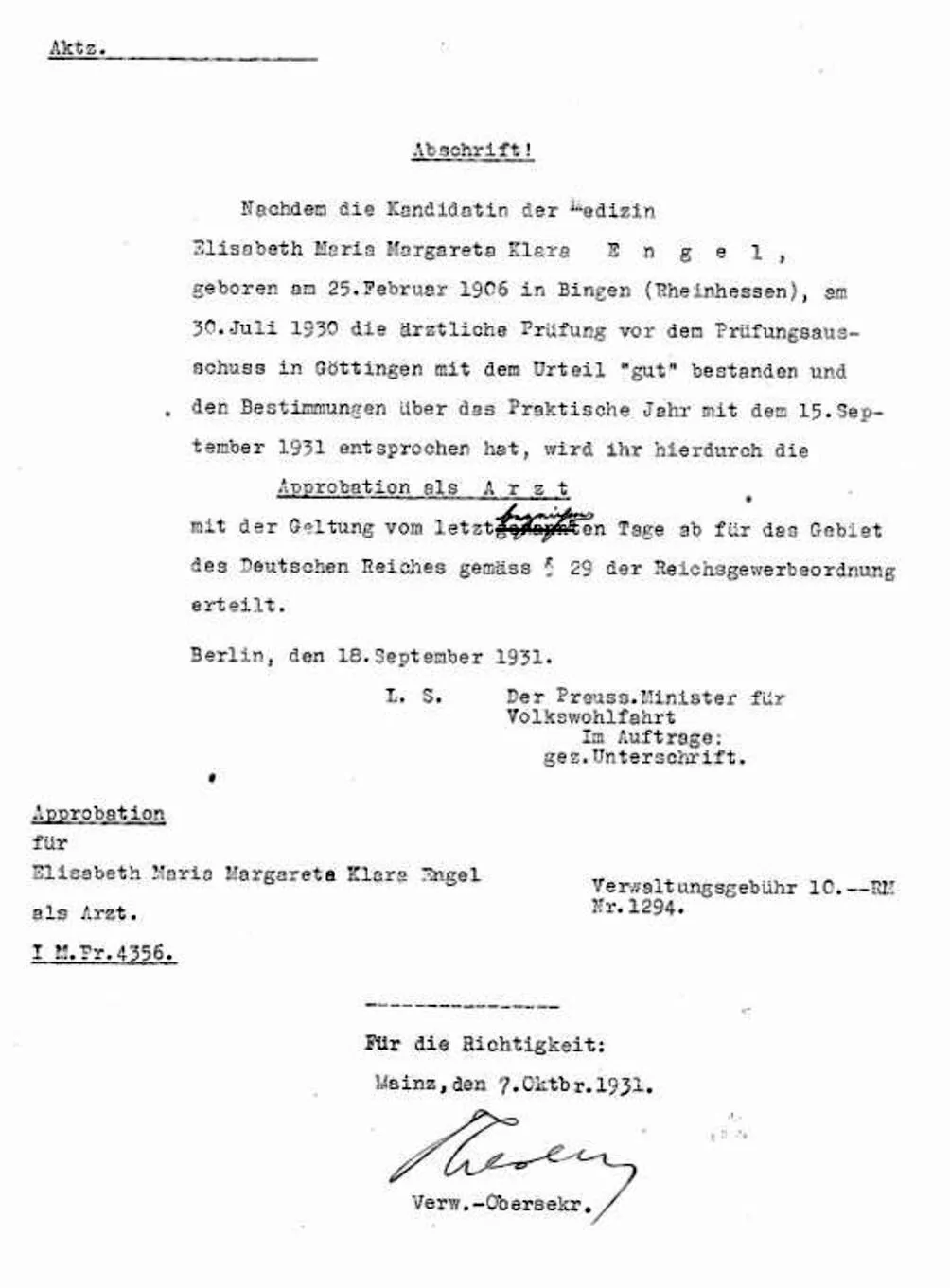
Abschrift Erteilung der Approbation Elisabeth Maria Margarete Klara Engel, 1931^a^. (Mit freundl. Genehmigung. ^a^StadtA Mainz, Bestand: 90/Zugang vor 1962, Engel, Elisabeth [Elsbet], geb. 25.02.1906.)

In der Folge (1930) wirkte Elsbet Engel als Medizinalpraktikantin in der Inneren Abteilung des Städtischen Krankenhauses Mainz.^7^ Das Praktische Jahr war Teil der Ausbildung und musste vor Erteilung der Approbation am 18. September 1931, absolviert werden (Abb. 5).

Die junge Ärztin blieb auch danach zunächst der heimatnahen Mainzer Klinik als Assistenzärztin in der Inneren Abteilung erhalten.^8^ Ihre Entscheidung für die Innere Medizin entsprach dabei durchaus zeitgenössischen Spezialisierungsmustern von Ärztinnen: Anfang der 1930er-Jahre waren 45 % der Fachärztinnen in der Pädiatrie, 14,5 % in der Gynäkologie und lediglich 7,5 % in der Inneren Medizin tätig [2, 36].

Wissenschaftlich beschäftigte sie sich zu diesem Zeitpunkt mit hereditären degenerativen Muskelerkrankungen, wie ihre gemeinsame Publikation mit ihrem damaligen Kollegen Godehard Goralewski [12], dem späteren Dozenten für Luftfahrtmedizin und Oberarzt der Poliklinik an der medizinischen Akademie Düsseldorf (1934–1937), „Zur Behandlung der Dystrophia musculorum progressiva mit Adrenalin und Pilokarpin“ [13], aus dem Jahr 1932 belegt.

Während ihrer Assistenzarzttätigkeit lernte sie auch ihren späteren Ehemann Werner Forßmann (1904–1979) kennen, der 1956 für seine Herzkatheterisierung mit dem Nobelpreis ausgezeichnet werden sollte. Dieser hatte zu dieser Zeit eine Assistentenstelle in der Chirurgie inne [4, 9, 10, 16, 17, 31, 32, 35]. In seiner Autobiographie „Selbstversuch. Erinnerungen eines Chirurgen“ (1972) beschreibt er die erste Begegnung mit ihr wie folgt:

„Plötzlich öffnete sich die Tür, und die diensthabende Ärztin der Inneren Abteilung kam herein. Ich wollte gerade aufstehen, um mich vorzustellen, als das Telefon läutete […]. Als wir uns nachher gegenseitig vorstellten, war mir in diesem Augenblick mit nachtwandlerischer Sicherheit klar, dass ich diese Hand nie wieder loslassen würde“ [9, S. 136]

Um ihre internistische Spezialisierung weiter voranzutreiben bewarb sich Engel in Greifswald für eine Stelle in der Inneren Klinik, die sie im Oktober 1932 auch hätte antreten können [33]. Aufgrund des Heiratsantrags, den ihr Werner Forßmann machte, verzichtete sie jedoch auf eigene Karriereambitionen [9, 33]. In seinem „Selbstversuch“ heißt es hierzu:

„Fräulein Engel, warum wollen Sie eigentlich nach Greifswald gehen? Sie können doch ebenso gut hier in Mainz bleiben – Wie denken Sie sich das denn, ich habe ja hier schon meine Stelle aufgegeben. […] Ich raffte meinen Mut zusammen: ‚Da wüsste ich schon etwas für Sie. Werden Sie doch einfach meine Frau!‘“ [9, S. 142]

Nach der Verlobung mit Forßmann verblieb Engel in Mainz als Assistenzärztin (ab 15. Oktober 1932), jedoch nun nicht mehr an der Inneren Klinik, sondern am Pathologische Institut des Städtischen Krankenhauses.^9^ Ihr Haupttätigkeitsfeld bestand in Laborarbeiten [9, 33].

## Dozentur an der Mainzer Frauenarbeitsschule

Parallel zu ihrer Assistenzarzttätigkeit übte Elsbet Engel seit dem 24. März 1933 eine nebenamtliche Dozentur für das Fach der Gesundheitspflege an der 1896 gegründeten Mainzer Frauenarbeitsschule aus.^10^ Diese Frauenarbeitsschule, eine Art Volkshochschule für Frauen und Mädchen, hatte ihren Sitz im Haus Emmeransstraße 41 und am Karmeliterplatz. Geboten wurde ein breites Spektrum an Kursen, aber auch die Möglichkeit zu einer Berufsausbildung – sei es eine Ausbildung zur Schneiderin, Handarbeitslehrerin oder seit 1906 auch das Berufsbild der damals sog. Kindergärtnerin und Kinderpflegerinnen [20].^11^ Mit Anbeginn der NS-Zeit widmete sich die Schule vor allem der „Erziehung zur deutschen Frau und Mutter“ [38]. Gemäß dem Duktus vom Arzt als Erzieher ist anzunehmen, dass Engel neben gesundheitshygienischem Unterricht der potentiellen Mütter auch in der Berufsgruppe der Kinderpflegerin eingesetzt wurde. So gehörte ab 1933 die Belehrung über die „schicksalhafte Bedeutung der Vererbung einerseits, die Verantwortung des Erziehers andererseits“ [38 S. 129] zum Ausbildungsspektrum der jungen Kinderpflegerinnen. „Die Erkenntnis, daß die Kinderpflegerin insbesondere auch auf dem Lande in starkem Maße in der Lage sein muß[te], gesundheitspflegerisch tätig sein zu können, [war] […] bestimmend für die Behandlung dieser Fragen auf breiter Grundlage“ [38, S. 129].

Die Erziehungswissenschaftlerin Ina Kaul (2025) von der Universität Kassel bemerkt dazu einordnend: „Soziale Arbeit mutierte zur ideologisch ausgerichteten ‚Volkspflege‘ am arischen, erbgesunden, leistungsfähigen und politisch angepassten Volksgenossen und dessen Familie“ [19, S. 175].

Obsolet zu sagen, dass eine entsprechende ideologische Prägung auch von Seiten der Dozentenschaft verlangt wurde [20]. So war Elsbet Engel seit Sommer 1933 Mitglied der NS-Frauenschaft geworden.^12^

## Heirat und Umzug nach Berlin

Elsbet Engels spätere urologische Fachspezialisierung ist eng mit der Vita ihres Ehemanns verknüpft; Informationen erhalten wir fast ausschließlich aus seiner Autobiographie, was aus quellenkritischer Sicht als schwierig zu bewerten ist, da Parallelüberlieferungen fehlen. Daraus geht hervor, dass die Eheschließung mit Werner Forßmann Anfang Dezember 1933 in der Altmünsterkirche in Mainz, zu einer jähen Zäsur ihrer ärztlichen Tätigkeit führte. Grund hierfür war die seit 1879 geltende sogenannte „Zölibatsklausel“. Jene war Ausdruck der damaligen gesellschaftlichen und arbeitsrechtlichen Vorstellungen, wonach verheiratete Frauen ihre Rolle vorrangig in der Familie und als Mütter sahen und nicht in der Erwerbstätigkeit und reglementierte so ein Ausscheiden von verheirateten Frauen aus Positionen des öffentlichen Dienstes [36].

Auch für akademische Berufe wirkten sich die nationalsozialistischen Bestrebungen zur Begrenzung des sogenannten „Doppelverdienertums“ aus. Vor dem Hintergrund des Leitbildes des männlichen Familienernährers wurden insbesondere verheiratete Frauen in ihrer Erwerbstätigkeit eingeschränkt oder bei Einstellungen und beruflicher Anerkennung benachteiligt [11]. Ergänzend wirkten Verwaltungspraxis und berufsständische Regelungen darauf hin, verheiratete Frauen aus bestimmten Berufsfeldern zurückzudrängen [1, 2, 18, 36, 41]. Auch Ärztinnen blieben trotz des freiberuflichen Charakters des Arztberufs nicht von geschlechterpolitischen Vorstellungen zur Begrenzung weiblicher Erwerbstätigkeit unberührt, die sich u. a. auf berufliche Beschäftigungs- und Entwicklungsmöglichkeiten auswirkten [1, 2, 11, 18, 36, 37, 41, 42]. Im Fall von Elsbet Engel führte dies dazu, dass sie ihre Stelle am Pathologischen Institut des Städtischen Krankenhauses in Mainz im Oktober 1933 unter Verweis auf ihre bevorstehende Eheschließung aufgab; das Arbeitsverhältnis endete im Dezember desselben Jahres.^13^

 Im Herbst 1933 wurde unter der Leitung des Urologen Karl Heusch (1894–1986), einem Schüler von Otto Ringleb (1875–1946), die erste und einzige urologische Abteilung in einem städtischen Krankenhaus in Berlin eröffnet. Dies markierte einen entscheidenden Schritt in der Etablierung der Urologie als eigenständiges medizinisches Fachgebiet im deutschsprachigen Raum, das erst neun Jahre zuvor mit der Einführung der Facharztprüfung für Urologie anerkannt worden war [28]. Heusch, ein Pionier auf diesem Gebiet, bot dem angehenden Urologen Werner Forßmann eine Oberarztstelle im renommierten Rudolf-Virchow-Krankenhaus an, was die Bedeutung dieser neu gegründeten Abteilung weiter unterstrich [21, 22]. Infolge des Umzugs nach Berlin fand sich Elsbet Forßmann in einer neuen Lebenssituation wieder und fühlte sich ohne eine eigene ärztliche Berufsausübung unvollständig. Um ihrer beruflichen Ader Ausdruck zu verleihen, nahm sie eine Volontärposition in der urologischen Fachabteilung des Rudolf-Virchow-Krankenhauses an, wo sie bis zum Sommer 1934 tätig war. Wie aus den Lebenserinnerungen ihres Mannes hervorgeht, umfassten ihr Aufgaben u. a. die Assistenz bei Nierenoperationen, wobei sie die Gelegenheit hatte, umfassende praktische Erfahrungen zu sammeln und schätzte die Vielfalt der urologischen Eingriffe: „Ich hatte geglaubt, immer wieder Nieren zu operieren, wäre sehr langweilig. Aber du machst es immer irgendwie anders und neu.“ [9, S. 159]. So scheinen die Kenntnisse die sie im Rahmen der „Berliner Urologenschule“ erworben hat, eine entscheidende Rolle in ihrer späteren Karriere als eine der ersten Urologinnen im deutschen Sprachraum spielten zu haben [9, 33]. Hier wurde der Grundstein von Elsbet Forßmanns urologischer Fachausbildung gelegt [33].

Ab 1936 arbeitete Werner Forßmann als Facharzt für Urologie in der Funktion eines chirurgischen Oberarztes zunächst unter Professor Albert Fromme (1881–1966) in Dresden und ab 1937 im Berliner Krankenhaus Moabit. [4, 16]. Für diesen Zeitraum sind keine weiteren ärztlichen Tätigkeiten für Elsbet Forßmann belegt, im Reichsmedizinalkalender von 1937 wird nur noch ihr Ehemann Werner geführt [8].

Da zwischen 1934 und 1943 die Geburten aller 6 Kinder der Familie – Klaus (1934), Knut (1936), Jörg (1938), Wolf-Georg (1939), Bernd (1940) und Renate (1943) – erfolgten, kann angenommen werden, dass Elsbet Forßmann in diesem Zeitraum überwiegend mit familiären Aufgaben und der Erziehung der Kinder beschäftigt war [33].

## Zweiter Weltkrieg

Zu Beginn des Zweiten Weltkrieges 1939 wurde Werner Forßmann als Sanitätsoffizier eingezogen [4, 35]. Seine Ehefrau verblieb vorerst mit den Kindern in Berlin. Nachdem die gemeinschaftliche Wohnung ausgebombt wurde, zog die Familie 1943 nach Wambach im Schwarzwald, wo ihre Elsbets Mutter lebte. Nachdem sie einige Zeit bei der Heuernte half, wurde Forßmann aufgrund des kriegsbedingten Ärztemangels gemeinsam mit einer aus Freiburg stammende Ärztin als Hilfskassenärztinnen verpflichtet [9, 33].

Jessica Peter vermutet, dass die Ausübung ihrer hausärztlichen Tätigkeit nach Kriegsende dazu führte, dass die Familie von Maßnahmen der sogenannten Rückführung der Evakuierten ausgenommen blieb und ihren Wohnsitz in der Gemeinde Wies beibehalten konnte [33].

Die landärztliche Tätigkeit beschreibt ihr Ehemann in seinen Memoiren in den Worten seiner Frau als durchaus ambivalent: „Die Praxis ist schrecklich, weil die Leute so nett sind. […] Komme ich in ein Haus, muß ich erst einen großen Kirsch trinken, beim Rezeptschreiben noch einen und beim Verabschieden den dritten. Nach 3 Krankenbesuchen bin ich fix und fertig […]“ [9, S. 278]. Darüber hinaus sei angesichts der fehlenden Privatpatienten finanziell wenig ertragreich, denn die Patienten zahlten zumeist in Naturalien [9], was in der unmittelbaren Nachkriegszeit jedoch bestimmt eine bessere Versorgung mit Lebensmitteln sicherstellte, als sie in großen Städten bestanden hätte. Werner Forßmann selbst überbrückte die Zeit der Entnazifizierung, in der er als ehemaliges NSDAP-Mitglied mehrere Jahre Berufsverbot hatte [31, 32]^14^, indem er seiner Frau in ihrer privaten Praxis zuarbeitete, die als „unbelastet“ entnazifiziert worden war [16, 31, 32, 40].^15^ Und das obwohl sie in ihrer Personalakte aus Mainz angab zumindest Mitglied in der NS-Frauenschaft gewesen zu sein und somit die Schulung und Erziehung im Sinne der NS-Gesundheitspolitik von Frauen nicht nur beeinflussen, sondern diese auch selbst potentiell mitgestaltet hatte.

1948 zog die Familie nach Wies um, noch im selben Jahr stufte die Spruchammer der französischen Besatzungsmacht Werner Forßmann als „Mitläufer“ ein [10, 16, 31, 32, 40].^16^ Gemeinsam mit ihrem Ehemann betrieb Elsbet Forßmann dann eine Allgemeinpraxis im Erdgeschoss eines Gasthofs, die Gaststube fungierte als Wartezimmer [9, 33, 40].

Ob dort auch urologische Erkrankungen behandelt wurden, lässt sich anhand der Quellen nicht eindeutig nachweisen, erscheint jedoch plausibel. Belegt sind hingegen zahnärztliche Behandlungen, die aufgrund der rund 20 km entfernten nächsten Zahnarztpraxis durchgeführt wurden [9, 33].

Im 1950 kann das Ehepaar dann auf Vermittlung der Landesärztekammer Koblenz eine frei gewordene Praxis in Bad Kreuznach sowie 18–20 Belegbetten in den „Kreuznacher Diakonie-Anstalten“ übernehmen [40]. Elsbet Forßmann assistierte auch hier ihrem Mann „beim Operieren, in der Sprechstunde und bei den Röntgenuntersuchungen nachmittags in den Diakonieanstalten“ [40] und konnte darüber, wie sich ihr Sohn Jörg Forßmann der Architekt und Stadtplaner in einer Reportage über den Vater in der SWR Landesschau Rheinland-Pfalz aus dem Jahr 2019 erinnert, ihre Expertise im Fach Urologie weiter ausbauen [40].

**Abb. 6:**
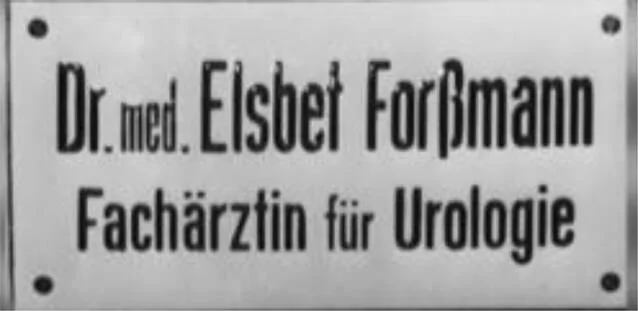
Praxisschild Elsbet Forßmann (Bad Kreuznach). (DGU-Archiv, Repro Keyn, mit freundl. Genehmigung)

In der Tat umfasst ihre Tätigkeitsspektrum der Urologie. Hierzu gehörten neben Nierenoperationen ebenfalls onkologische Eingriffe an der Harnblase sowie die Behandlung der Prostatahyperplasie und postgonorrhoischen Harnröhrenstrikturen [9, 33]. Am 22.09.1954 erhielt Elsbet Forßmann die Anerkennung als Fachärztin für Urologie und zählte somit neben Elisabeth Schmitz aus Wuppertal-Barmen in der Nachkriegszeit zu eine der ersten Fachärztinnen für Urologie ([33]; Abb. 6).

Sieben Jahre später – inzwischen hatte er gemeinsam mit André Frédéric Cournand (1895–1988) und Dickinson Woodruff Richards (1895–1973) den Nobelpreis für Physiologie oder Medizin erhalten – übernahm Werner Forßmann die Leitung der Chirurgie des Evangelischen Krankenhauses in Düsseldorf, wo er bis zu seinem Ruhestand 1970 seine Wirkungsstätte haben sollte [4, 9, 17, 31–33]. Mit dem Umzug nach Düsseldorf legte Elsbet Forßmann ihre ärztliche Tätigkeit nieder. Den Ruhestand verbrachte das Ehepaar in Wambach im Schwarzwald. Elsbet Forßmann starb 1993 im Alter von 87 Jahren [33].

## Schluss

Die Erinnerung an Elsebt Forßmann wurde lange Zeit vor allem durch die fachkulturelle Erinnerung an ihren Ehemann, den Nobelpreisträger überstrahlt [22]. Ihre Wiederentdeckung erfolgte dabei 2008 im Rahmen der Dissertation von Jessica Peter zu den ersten Urologinnen in Deutschland [33].

Welche Schlüsse können wir aus der Biografie Elsbet Forßmann ziehen? Obwohl für sie aufgrund der finanziellen Möglichkeiten ihrer Familie der Erwerb des Abiturs und die Aufnahme eines Studiums möglich waren, deuten diese Aspekte aber gleichzeitig klar weiterhin auf die sozioökonomische Exklusivität einer akademischen Ausbildung im frühen 20. Jahrhundert; so wie wir es bereits aus den Lebensgeschichten anderer früher Urologinnen wie z. B. Dora Gerson kennen, hin [22, 30, 33]. Im Sinne der Intersektionalität müssen diese Hürden also weiterhin für viele Frauen als noch stärker bremsend eingeschätzt werden als für Männer [1, 2, 11, 26–30].

Mit Blick auf die Zeit des Nationalsozialismus wird deutlich, dass sowohl ihr Mann Werner, als auch sie selbst, sich nicht im Widerstand zum NS-System positioniert haben, sondern NS-Organisationen beitraten und politisch mit dem System sympathisierten [32]. So prägten bereits in der Zeit vor 1933 Entwicklungsstörungen und Vererbungsfragen Elsbet Forßmanns wissenschaftliches Interesse. Dieses war somit anschlussfähig an den Eugenikdiskurs der Zeit. Des Weiteren wurde Elsbet Forssmann Mitglied in der NS-Frauenschaft und unterrichtete an einer Schule, an der Frauen in frauenkonformen Berufen, ergänzend mit NS-Gesundheitsaspekten, ausgebildete wurden. Mit der Geburt ihrer 6 Kinder zwischen 1934 und 1943 erfüllte sie auch in ihrem Privatleben das, was im NS von ihr bevölkerungspolitisch erwartet wurde. Gleichzeit verhinderte die Nichtmitgliedschaft in der NSDAP im Gegensatz zu ihrem Ehemann ein Berufsverbot in der Nachkriegszeit. Somit war sie es, die in den Jahren der Entnazifizierung von Werner Forßmann die Rolle der Ernährerin ausübte und ihrem Mann die Möglichkeit bot, in ihrer Landarztpraxis durch (formelle) Zuarbeiten diese Zeit zu überbrücken. Somit kann man rückblickende festhalten: Elsbet Forßmann war zeitweise die Chefin des zukünftigen Nobelpreisträgers.

## Data Availability

Alle dieser Arbeit zugrunde liegenden Daten sind in diesem Artikel enthalten.

## Footnotes

1 Auskunft Standesamt Bingen am Rhein vom 18.08.2025.

2 Das Durchschnittsentgelt im Deutschen Reich zu dieser Zeit lag bei lediglich 1469 RM [3]. Das Schulgeld belief sich jedoch in den Jahrgangsstufen 10 bis 7, die Zählung der Klassen begann oben, auf 10 bis 7 jährlich 66 Mark, in den Klassen 6 bis 4 auf 84 Mark, in den Klassen 3 bis 1a dann auf 120 Mark und in der 1b, der sog. Seminarabteilung, auf jährlich 150 Mark [42].

3 StadtA Mainz, Bestand: 90/Zugang vor 1962, Engel, Elisabeth (Elsbet), geb. 25.02.1906. Im Universitätsarchiv Jena ist lediglich ein Eintrag von Elsbet Engel in der Studentenkartei (1916–1936) überliefert. Aus diesem geht hervor, dass sie sich am 29.04.1925 in Jena für das Studium der Medizin immatrikuliert hat und zum 20.10.1927 exmatrikuliert wurde. Auskunft Schreiben Universitätsarchiv Jena vom 09.12.2024.

4 StadtA Mainz Bestand, 90/Zugang vor 1962, Engel, Elisabeth (Elsbet), geb. 25.02.1906; UA Göttingen, Bestand: Abgangszeugnis 1930 Nr. 728; UA München, Bestand: Stu-Kart I Engel.

5 UA Göttingen, Bestand: Med Prom 1553.

6 UA Göttingen, Bestand: Abgangszeugnis 1930 Nr. 728.

7 StadtA Mainz, Bestand: 90/Zugang vor 1962, Engel, Elisabeth (Elsbet), geb. 25.02.1906. Bleker (2011a) und Peter geben jedoch an, dass Engel an der Kinderklinik gewirkt haben soll [1, 33]. Das ist der Mainzer Personalakt jedoch nicht zu entnehmen.

8 Ihre Assistenzzeit verbrachte sie ab dem 1. Januar 1931 zuerst als Medizinalpraktikantin an der Inneren Klinik des Städtischen Krankenhauses Mainz, um dort ab dem 10. Oktober 1931 zur Assistenzärztin I Klasse aufzusteigen. StadtA Mainz, Bestand: 90/Zugang vor 1962, Engel, Elisabeth (Elsbet), geb. 25.02.1906.

9 StadtA Mainz, Bestand: 90/Zugang vor 1962, Engel, Elisabeth (Elsbet), geb. 25.02.1906.

10 StadtA Mainz, Bestand: 90/Zugang vor 1962, Engel, Elisabeth (Elsbet), geb. 25.02.1906.

11 Diese ein- bis eineinhalb-jährigen Kurse endeten mit einer staatlichen Prüfung. Mainz war zu dieser Zeit die einzige Stadt im hessischen Raum, die eine solche Ausbildung anbot [42].

12 StadtA Mainz, Bestand: 90/Zugang vor 1962, Engel, Elisabeth (Elsbet), geb. 25.02.1906.

13 StadtA Mainz, Bestand: 90/Zugang vor 1962, Engel, Elisabeth (Elsbet), geb. 25.02.1906.

14 StaatsA Freiburg, Betand: D 180/2 Nr. 201710.

15 StaatsA Freiburg, Bestand: D 180/2 Nr. 57254.

16 StaatsA Freiburg, Bestand: F 202/2 Nr. 4236.
